# Video Game to Attenuate Pandemic-Related Stress From an Equity Lens: Development and Usability Study

**DOI:** 10.2196/36820

**Published:** 2022-05-12

**Authors:** Nadia Minian, Anika Saiva, Allison Gayapersad, Rosa Dragonetti, Catherine Proulx, Patricia Debergue, Julia Lecce, Sarwar Hussain, Eric Desjardins, Peter Selby

**Affiliations:** 1 Nicotine Dependence Service Centre for Addiction and Mental Health Toronto, ON Canada; 2 Department of Family and Community Medicine University of Toronto Toronto, ON Canada; 3 Campbell Family Mental Health Research Institute Centre for Addiction and Mental Health Toronto, ON Canada; 4 Institute of Medical Science Faculty of Medicine University of Toronto Toronto, ON Canada; 5 Medical Devices Research Centre National Research Council Canada Boucherville, QC Canada; 6 Department of Psychiatry University of Toronto Toronto, ON Canada; 7 Dalla Lana School of Public Health University of Toronto Toronto, AB Canada

**Keywords:** video games, cognitive behavioral therapy, usability study, self-care, digital health, technological infrastructure, video game development, user engagement, user perception, COVID-19, Mental health, mental health care system, depression, anxiety, digital therapy

## Abstract

**Background:**

The emergence of the novel coronavirus (COVID-19) has introduced additional pressures on an already fragile mental health care system due to a significant rise in depression, anxiety, and stress among Canadians. Although cognitive behavioral therapy (CBT) is known to be an efficacious treatment to reduce such mental health issues, few people have access to CBT in an engaging and sustainable manner. To address this gap, a collaboration between the Centre for Addiction and Mental Health (CAMH) and the National Research Council of Canada (NRC) developed CBT-based self-led, online, clinician-tested modules in the form of a video game, named Legend of Evelys, and evaluated its usability in the attenuation of a COVID-19–related increase in stress.

**Objective:**

We here present the conceptualization and design of new self-care modules in the form of a video game, its implementation in a technological infrastructure, and inclusivity and privacy considerations that informed the development. A usability study of the modules was performed to assess the video game’s usability, user engagement, and user perceptions.

**Methods:**

The development of the video game involved establishment of a technology infrastructure for secure implementation of the software for the modules and a clinician-led assessment of the clinical utility of these modules through two “whiteboard” sessions. The usability study was informed by a mixed methods sequential explanatory design to evaluate the intervention of the mobile app through two distinct phases: quantitative data collection using in-app analytics data and two surveys, followed by qualitative data collection by semistructured interviews.

**Results:**

A total of 32 participants trialed the app for 2 weeks. They used the video game an average of six times and rated the game as “good” based on the Systems Usability Scale score. In terms of stress reduction, the study demonstrated a significant difference in the participants’ Perceived Stress Scale score at baseline (mean 22.14, SD 6.187) compared with that at the 2-week follow-up (mean 18.04, SD 6.083; t_27_=3.628, P=.001). Qualitative interviews helped participants identify numerous functionality issues and provided specific recommendations, most of which were successfully integrated into the video game for future release.

**Conclusions:**

Through this collaboration, we have established that it is possible to incorporate CBT exercises into a video game and have these exercises adopted to address stress. While video games are a promising strategy to help people with their stress and anxiety, there is a further need to examine the real-world effectiveness of the Legend of Evelys in reducing anxiety.

## Introduction

### Background

Stress levels and anxiety are elevated at a population level during crises. The impact of COVID-19 being declared a pandemic predictably had a negative effect on the mental health of many Canadians. Researchers worldwide have shown that the mental health of the general population has deteriorated since the onset of the pandemic, with significant rises in depression, anxiety, and stress [1-3]. Approximately 19% of Canadians reported experiencing moderate to severe anxiety and 19% reported feeling depressed occasionally since the start of the pandemic [4]. Even prior to the pandemic, anxiety disorders were among the most prevalent mental health problems in Canada [5], having a profound negative impact on life expectancy, quality of life, and health care utilization [6,7].

Cognitive behavioral therapy (CBT) has been shown to be an efficacious treatment to reduce anxiety [8-10], stress [11], and acute stress disorder [11,12]; however, few people have access to this treatment [13]. In efforts to make CBT more accessible, many Canadian institutions offer CBT online, which has been shown to be effective [11]. However, when CBT is provided online, there are more problems with adherence (completing the intervention to the extent that the developers intended it to be used) and engagement (the extent, both in terms of time and frequency, that participants visit the website/app) [14]. Video games are a promising solution to overcome many of these barriers [15], as they can provide a way of motivating and engaging users [16] and can promote adherence to treatment [16]. Video games have been found to be beneficial in reducing stress and anxiety among children, adults, and older adults, even in the instances of single or short sessions of play [17,18]. In 2020 alone, 35% of Canadians reported playing online video games and 10% reported playing for more than 10 hours a week [19]. A recent study found that playing video games has a positive effect on players’ perceived well-being during the COVID-19 pandemic [18]. Furthermore, 84% of Canadians reported using smartphones for personal use daily [19]. Given that in the past decade smartphones have become a common possession, irrespective of gender, race/ethnicity, or socioeconomic status [20], disseminating CBT interventions via smartphones allows for greater, more equitable accessibility regardless of geographic and economic restrictions [21].

In 2020-2021, the National Research Council of Canada (NRC) and the Centre for Addiction and Mental Health (CAMH) developed a video game to help people reduce their stress in the context of COVID-19. The video game is a mobile role-playing game based on CBT principles, which supports users in building the necessary skills to take care of their mental health during a pandemic. The version of the video game used in this study was in an early development stage with a short storyline and was limited to four CBT features.

In this manuscript, we describe the development of the video game as well as the results of a usability study, which assessed the video game’s usability, user engagement, and user perceptions*.* We situate this work within the Medical Research Council guidelines for complex intervention research [22].

### Development

As part of the development of this video game, two whiteboard sessions with eight clinicians were held virtually at the CAMH. The whiteboard sessions took place in two 1.5-hour meetings (July 22, 2020, and August 12, 2020). The main purpose of the sessions was for mental health experts to explore the platform, develop CBT and other evidence-based interventions and content, and provide creative feedback on the storyline and video game. The meetings with clinicians generated effective feedback, and encouraged collaboration and knowledge-sharing that were integral to the conceptualization and design of the video game. The first meeting focused on the demonstration of the first idea for the video game and elicitation of the clinicians’ input. The second meeting focused on CBT content/activities planning and storyline development.

Initial recommendations from clinicians after viewing the demonstration of the prototype was that the video game should complement existing CBT resources. The team agreed that the context and characters included in the video game should be accessible, inclusive, and culturally diverse, and offer choice to the user. Discussion and feedback were provided on the multiple CBT strategies and other interventions or activities that would be useful to include, such as cognitive restructuring, mindfulness, and journaling. Other suggestions included to focus on positive feedback, conduct a needs and readiness assessment, create incentives/rewards, link exercise results to other exercises, and have peer-to-peer support built in/discussion board.

Prior to the second whiteboard session, the feedback from the first whiteboard session was consolidated and incorporated into another version of the game. The second whiteboard session included a demonstration of the updated features. The clinical team shared CBT strategies that worked in practice with their patient population, and brainstormed on the CBT tools that could be included in the video game and may be easier to gamify. The developers included a novel feature, a “cognitive monsters activity,” which the team agreed was appropriate for the video game but further thought it would be important to provide some psycho-education and positive feedback on this cognitive monster feature. Cognitive monsters represent unhelpful ways of thinking (formerly known as cognitive distortions). The team recommended a “dashboard” feature based on the CBT model to provide a rationale and cohesive narrative/framework (storyline) to make the app easier to follow for users. Other suggestions included: integration into the health care system, allow customizable affirmations, and prompts for goal setting. In general, the team liked how the content was presented but expressed concern about reaching the wider population.

After the two whiteboard sessions, two CAMH clinicians (RD and PS) outlined the CBT features that ended up being included, and the NRC (CD) came with creative ideas on how to gamify them in the COVID-19 context.

This video game, developed in Unity, involves a fantasy storyline centered on the experience of negative emotional states among citizens in a fictitious place, intended to mirror the experience of the COVID-19 pandemic and negative mental health outcomes. Unity is an industry-standard cross-platform game engine, which holds the largest market share for mobile game development. Players of the video game can customize their own avatars via a customization “closet,” where users can pick a skin color, choice of pronouns, hairstyle, and color and type of clothing, and then proceed through adventures in the village. The video game includes a storyline before entering the game screen, which narrates the concept behind the game. The video game allows for interactions between characters in the environment and dialog boxes appear prompting the user to engage in specific CBT-related exercises (see Multimedia Appendix 1).

### Strategies

#### Overview

We included core CBT strategies such as cognitive restructuring, journaling, and tracking behaviors, as well as some additional evidence-based strategies such as mindfulness exercises and community support.

#### Cognitive Restructuring

An educational module was included, which intended to teach the user how to identify unhelpful ways of thinking (biased or distorted thoughts) by encountering “cognitive monsters” [23].

Exercises that would typically be proposed to participants in a cognitive behavioral program were transformed into confrontations with virtual monsters. Through multiple battles with the “cognitive monsters,” users learned about cognitive restructuring strategies. Monsters “attacked” the user with a statement, and the user had to correctly respond to that statement in order to deflect the attack. Monsters were transformed into tame creatures when participants responded to the monsters with more balanced statements (see Multimedia Appendix 2).

#### Journaling and Goal-Setting (Behavioral Activation)

A journaling and behavior-tracking feature called the “blue book” was included in the design [24]. The journal allowed free-form entries, where users could reflect on their mental health and write down their thoughts. The goals module in the journal allowed users to set and check personal goals (free-form entry) or select from a list of predefined daily goals (daily activities).

#### Breathing Meditation (Mindfulness)

A guided breathing activity was included, named the “breathing sphere” [25]. The “breathing sphere” exercise guided the user through a paced breathing sequence with various customizable options for pace, duration, music, and art.

#### Community Support

The community support module enables interactions with characters who experienced mental health issues and suggests coping mechanisms.

Characters in the virtual environment of the video game embodied different user stories related to their experiences during the COVID-19 pandemic. Through conversations with the player, they presented their own mental health challenges and proposed coping strategies. These stories were inspired by the main issues identified in the COVID-19 National Survey Dashboard from May 2020 to December 2020 [26].

### Connectivity

Together, the CAMH and NRC articulated the necessary technological infrastructure parameters for hosting the video game app. This included a cloud-based online microservices RESTful interface and a database to collect anonymized usage analytics. The user service was used for login and user management. After login, usage data were sent via the user entry service. Later, administrative users fetched the data and received a JavaScript Object Notation (JSON) file. Working with personnel from the CAMH’s Informatics Department, an internal security analysis of the NRC modules was conducted as well as a third-party Privacy Impact Analysis and Threat Risk Analysis. Overall, no critical severity vulnerabilities were found within these infrastructures, which confirmed that the security controls and measures were properly implemented. All necessary security risk findings from these assessments were configured prior to the start of this study.

### Inclusivity

The need for an inclusive and accessible experience led the game design process along three axes: diversity, accessibility, and technological capacity. In addition to providing avatar customization, the game also featured a diverse cast of characters. Avatars and characters can be customized with four skin colors; custom hair color; multiple hairstyles, including hair coverings; and three choices of pronouns (male, female, and nonbinary). Accessibility features included high-contrast user interface elements, large fonts and buttons, and voice-over for dialogs. In addition, two navigation modes were available to provide alternate modes of moving the character: an on-screen joystick and a “click on destination” mode. The design team also validated that the game was usable with a variety of adaptive styluses.

In terms of technological capacity, care was taken to support older operating systems (minimum Android application programming interface level 19) so that usage of the game would not be limited to users with high-performance devices. This was a decision that needed to be taken at the very beginning of the design stage, as it determined the features and libraries available for development. In addition, the game did not require continuous connectivity, and data usage could be restricted to take into account the cost and possible unavailability of cell phone data in remote communities.

### Balanced Use and Privacy

Given the concern about the abusive use of video games and the potential vulnerability of our users, care was taken to avoid features that might cause users to overuse the game, such as random treasures with variable value (“loot boxes”). The game could be stopped at any time with no loss, and the core game loop was designed for short sessions.

Care was also taken to respect the sensitive nature of participants’ input into the game. The app did not ask for access to the device’s GPS, camera, microphone, contacts list, gallery, or any other feature that could breach confidentiality. In addition, all personal fields (diary, custom goals, personal thoughts and concerns) were encrypted on the device and were not included in the collected analytics data.

Future versions of the video game will incorporate additional features to encourage balanced play, such as pause prompts, rewards for coming back after a hiatus rather than punishment for breaking a streak, and features that require calendar time to advance to encourage frequent check-ins rather than long sessions.

## Methods

### Study Design

For this usability study, we utilized a mixed methods sequential explanatory design, which consisted of two distinct phases: quantitative data collection and analysis followed by qualitative data collection and analysis [27]. The qualitative (semistructured interviews) data help explain and put in context the quantitative results obtained in the first phase.

### Ethics Approval

The study was approved by the CAMH Research Ethics Board (111-2020).

### Participants and Recruitment

We used a purposive sampling approach with the aim of recruiting a diverse group of 40 participants from different ages and genders. Participants who were (1) 18 years or older, (2) owned an Android smartphone or tablet, (3) were willing to provide an email address, (4) were able to read and speak English, and (5) self-reported stress were eligible to participate in the study. Participants were excluded if they had any severe psychiatric illness that could impact the consent process.

We stratified our recruitment by age and gender identity. Our aim was to have representation from people of four different age groups (18-28 years, 29-39 years, 40-50 years, 50 years or older) and different gender identities. We aimed to recruit 10 participants in each of the age categories with representations from the different genders.

Participants were recruited using several cost-free strategies, including advertisements on the CAMH Nicotine Dependence Clinic website (a unit within the CAMH where the study was conducted), Kijiji, and Twitter. Interested participants emailed or called the research coordinator as instructed on the study advertisement and completed a screening call to assess eligibility. Eligible participants completed a consent discussion call with the research coordinator and were emailed a copy of the consent form to sign if they agreed to participate. All participants provided digitally signed informed consent prior to initiating the study.

### Study Procedure and Data Collection

After screening and consent procedures, participants completed an online baseline survey and were given access to the video game. Participants were asked to notify the research team once they had successfully downloaded and logged into the video game. A 15-minute check-in phone call was set up within the first week of the study, during which the participants could ask the research team any questions they had about the video game following their initial use. Two weeks after receiving access to the video game, participants completed an online follow-up survey and participated in a semistructured interview. The semistructured interview was conducted over the phone and lasted approximately 30 minutes. They were paid CAD $35 (approximately US $28) for completing all surveys and participating in the semistructured interview.

In the baseline assessment, participants completed measures of their sociodemographic characteristics, perceived physical and mental health status, substance use activities in the past month, and subjective stress levels. Subjective stress levels were assessed using the widely used Perceived Stress Scale (PSS-10) developed by Cohen Sheldon [28]. This 10-item validated tool has good internal reliability (Cronbach α=.78-.91) and is scored on a 5-point Likert scale ranging from 0 (never) to 4 (very often) [28,29]. Scores in the range of 0-13 are considered to indicate low perceived stress, scores in the range of 14-26 are considered moderate perceived stress, and scores in the range of 27-40 are considered high perceived stress.

The follow-up survey also collected the following information: (1) subjective stress levels using the PSS-10; (2) measures of usability of the video game, using the System Usability Scale (SUS) [30]; and (3) overall satisfaction and perceptions about the video game. The SUS is a 10-item measure assessing usability and user satisfaction with technology. The average SUS score for most technology is 68 at the 50th percentile. An SUS score greater than 68 is considered above average or “good,” whereas a score below 68 is considered below average or “poor” [30]. Overall satisfaction and perceptions were assessed using three 11-point Likert-scale questions in the survey: (1) overall satisfaction with the video game (0=not satisfied at all and 10=extremely satisfied), (2) burden of using the video game (0=not a burden at all and 10=extremely burdensome), and (3) the impact of the video game in reducing stress (0=did not help at all and 10=helped a lot).

All participants were scheduled for a 30-minute semistructured interview over the phone or a video conferencing system. Qualitative semistructured interviews were used to further elaborate on the usability of the video game and to explore how it could be improved. Usability was defined as the degree to which a program can be used easily, efficiently, and with satisfaction [31]. Participant ideas on how the video game could be improved were also explored. All interviews were audio-recorded and transcribed verbatim.

The CAMH server collected in-app analytics data on the use of the video game, such as the number of times participants opened the video game, number of times they used each component of the video game, and time spent engaged in those exercises. The app also collected general metadata about the device, such as the device model and operating system. Participants were asked to connect to a Wi-Fi network at the end of the 2 weeks of the study to transfer the analytic data onto the CAMH server.

### Analysis

Quantitative data were analyzed using SPSS (version 25). The SUS questionnaires in the follow-up survey was analyzed using scoring guidelines provided by the US Department of Health and Human Services [32]. Scores for each question were converted into a new number, summed, and then multiplied by 2.5 to obtain a score between 0 and 100, which informed the usability of the video game. Descriptive statistics are used to report sociodemographic characteristics, usability, and engagement. The PSS-10 was analyzed by first reversing scores for four of the positive items on the scale and then summing across all 10 items to obtain a score between 0 and 40 for each participant. A paired-samples *t* test was then used to compare subjective stress at baseline and after 2 weeks of trialing the video game. The data from the app logs were exported, tabulated, and analyzed using descriptive statistics with tools from Microsoft Excel.

Qualitative data analysis for the study involved an iterative, team-based process. Transcribed interviews were entered into NVivo 12 for qualitative data management and analysis. Interview transcripts were read multiple times by two research staff to achieve immersion prior to code development. One author (AG) crafted the initial codebook, and then AG and AS jointly coded the first four transcripts to refine the codebook and defined the codes through consensus. Two other transcripts were coded independently by AG and AS and the calculated interrater reliability was 80.2%. Remaining transcripts were independently coded either by AG or AS. Data were analyzed using thematic analysis [33]. A deductive approach was utilized to identify the coding scheme for the transcripts, which allowed for the development of codes corresponding to the components of usability and stress management.

We first analyzed our quantitative data, which helped to inform some of the probes we used when conducting the interviews. After we analyzed the interviews, we merged the results from both analyses (quantitative and qualitative) to provide a full interpretation of participants’ perceptions of the video game.

## Results

### Participants

**Figure 1:**
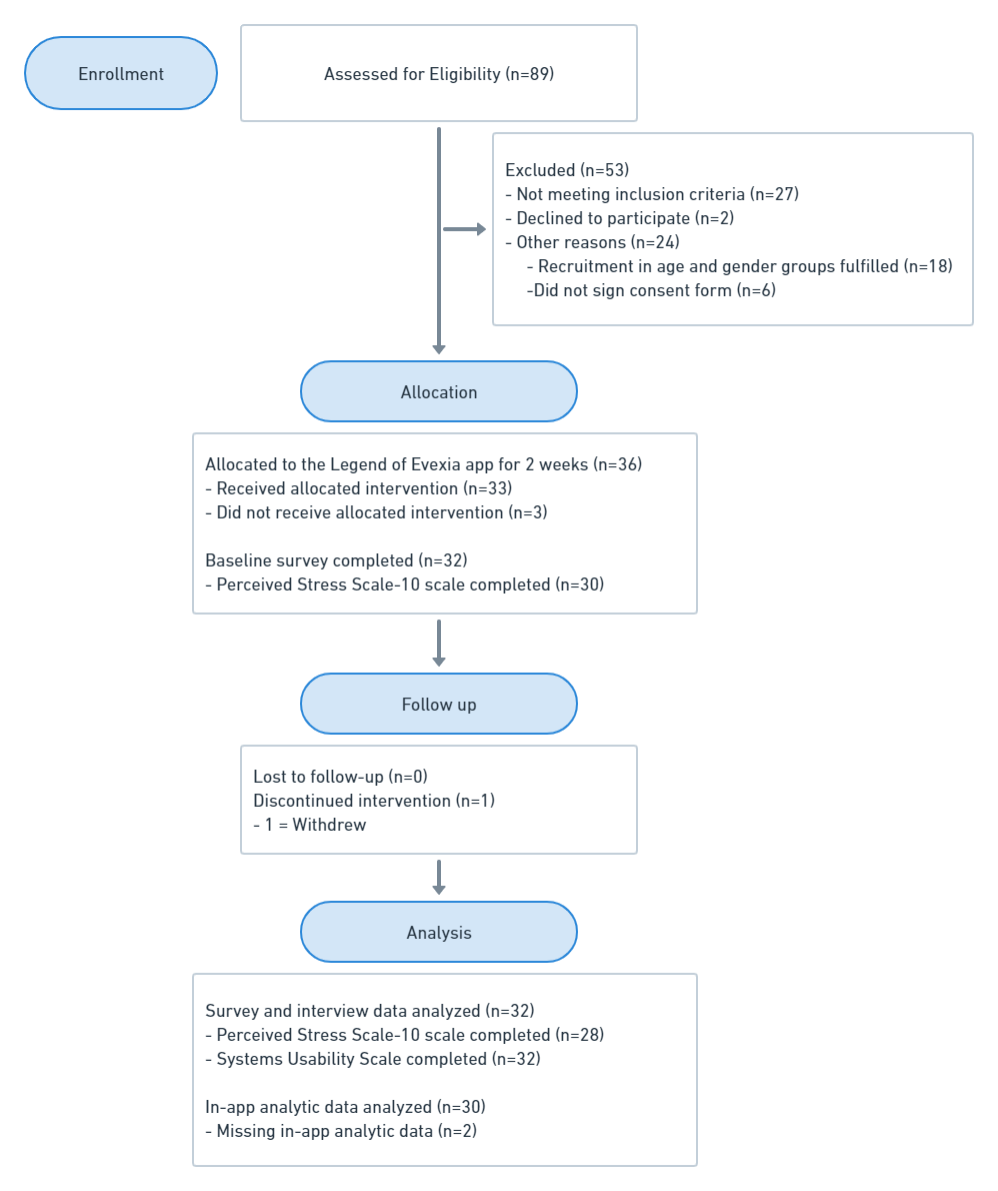
CONSORT flow diagram modified for non-randomized trial design.

A total of 185 individuals contacted the research team after coming across the advertisement for the study (Figure 1). Eighty-nine individuals were screened for eligibility and the remaining 96 individuals were unreachable after the initial contact. A total of 32 participants completed the study by trialing the app for 2 weeks and completing the baseline assessment, follow-up survey, and qualitative interview. Participants’ sociodemographic characteristics and descriptive statistics for all baseline variables are presented in Table 1.

**Table 1:** Baseline characteristics of participants (N=32).

Characteristics		Participants, n (%)
**Age (years)**		
	18-28	10 (31)
	29-39	10 (31)
	40-50	5 (16)
	51 or older	7 (22)
Most comfortable speaking English		32 (100)
**Birthplace**		
	Canada	20 (63)
	Outside Canada	12 (37)
**Racial or ethnic group**		
	East Asian (eg, Chinese, Japanese, Korean)	3 (9)
	South Asian (eg, Indian, Pakistani, Sri Lankan)	8 (25)
	Black-Caribbean (eg, Barbadian, Jamaican)	2 (6)
	Indian-Caribbean (eg, Guyanese with origins in India)	1 (3)
	Latin American (eg, Argentinean, Chilean, Salvadoran)	1 (3)
	White-European (eg, English, Italian, Portuguese, Russian)	4 (13)
	White-North American (eg, Canadian, American)	11 (34)
	Do not know/prefer not to answer	2 (6)
**Gender**		
	Woman-cisgender (assigned female at birth and has gender identities as woman)	17 (53)
	Man-cisgender (assigned male at birth and has gender identities as man)	14 (44)
	Nonbinary (gender identity does not align with a binary understanding of gender as man or woman)	1 (3)
**Sexual orientation**		
	Asexual	4 (13)
	Gay	1 (3)
	Heterosexual (straight)	24 (75)
	Pansexual	1 (3)
	Do not know/prefer not to answer	2 (6)
**Marital status**		
	Single (never married)	21 (66)
	Married or in a domestic partnership	8 (25)
	Separated or divorced	3 (9)
**Highest level of education**		
	High school	3 (9)
	Trade, technical or vocation school, apprenticeship training, or technical CEGEP^a^	2 (6)
	Diploma from community college, preuniversity CEGEP, or nonuniversity	8 (25)
	University degree	14 (44)
	Graduate degree (MSc, MBA, PhD, etc)	5 (16)
**Total family income (CAD $)^b^**		
	0-29,999	5 (16)
	30,000-59,999	13 (41)
	60,000-89,999	7 (22)
	90,000-119,999	3 (9)
	120,000-149,999	1 (3)
	150,000 or more	1 (3)
	Do not know/prefer not to answer	2 (6)
**Self-reported physical health status**		
	Excellent, very good, good	24 (75)
	Fair	4 (13)
	Poor	3 (9)
	Prefer not to answer	1 (4)
**Self-reported mental health status**		
	Excellent, very good, good	17 (53)
	Fair	12 (38)
	Poor	2 (7)
	Prefer not to answer	1 (3)
**Used tobacco products (cigarettes, chewing tobacco, cigars, etc) in the past month**		
	No	28 (88)
	Yes	4 (12)
**Used codeine or oxycodone in the past month**		
	No	32 (100)
	Yes	0 (0)
**Drank more than 5 alcoholic beverages on one occasion in the past month**		
	No	28 (88)
	Yes	4 (12)
**Used marijuana, cannabis, or hashish in the past month**		
	No	26 (81)
	Yes	6 (19)
**Perceived Stress Scale score**		
	Low stress (0-13 points)	5 (17)
	Moderate stress (14-26 points)	19 (63)
	High stress (27-40 points)	6 (20)
**Level of agreement with the following statement: “I am confident that I can reduce my stress using the video game”**		
	Very strongly agree or strongly agree	10 (31)
	Neither agree nor disagree	17 (53)
	Strongly disagree or disagree	5 (16)

^a^CEGEP: Collège d'Enseignement Général et Professionnel; public general and vocational college unique to the province of Quebec.

^b^1 CAD=US $0.79.

### Use of Video Game

In-app analytic/usage data were collected from 31 of the 32 participants. Analytic data from one participant were missing as they did not connect to a Wi-Fi network to transfer the data onto the server. Of the 31 participants, a few participants’ data on time spent within the video game were inaccurate due to app issues and were therefore omitted from the analysis where applicable.

During the 2 weeks participants were asked to use the video game, it was used a mean of 6 times (SD 8.75, range 1-47; n=31) and for an average of 21 hours (SD 33, range 0-147 hours; n=30).

In terms of the CBT features included in the video game, participants interacted with the characters in the video game an average 24 times (range 0-171) and spent an average of 10 minutes (SD 7.33; n=28) on the game. Participants completed exercises related to cognitive restructuring (wererabbits) an average of 10 times (range 1-48) for an average of 9 minutes (SD 11.25; n=28). The mindfulness exercise (the breathing sphere) was used an average of 4 times (range 0 -18) for an average of 10 minutes (SD 20.04; n=30). The average usage of the behavioral activation activities (journaling) was approximately 4 times (range 0-27) and participants spent an average of 40 minutes (SD 184.37; n=30) on these activities. On average, males and people over the age of 50 years used the video game more than people under the age of 50 years. The frequency of participant interaction with specific activities is shown in Table 2 by age and in Table 3 by gender.

**Table 2:** Analytic data of video game usage in sessions by age group.

Age group (years)	Participants, n	Total sessions of app usage		Journal sessions		Guided breathing activity sessions			Educational module sessions (ie, “cognitive monsters”)			Interaction with characters sessions	
		Mean (SD)	Range	Mean (SD)	Range	Mean (SD)	Range	Mean (SD)		Range	Mean (SD)		Range
18-28	10	2.9 (1.29)	1-4	1.1 (1.73)	0-5	1.5 (1.58)	0-4	4.5 (3.54)		0-10	8.7 (5.12)		1-16
29-39	9	5.6 (3.50)	2-12	2.6 (3.430)	3.430-11	4 (5.66)	0-18	14.6 (16.32)		0-54	19.7 (20.14)		0-62
40-50	5	4 (2)	2-6	3.4 (2.19)	1-7	4.8 (2.77)	2-9	10.6 (4.77)		5-17	18.4 (12.54)		8-40
50+	7	13.3 (17.1)	3-47	9 (10.02)	1-27	6 (5.45)	0-15	14.3 (13.3)		0-38	48.4 (57.06)		10-171

**Table 3:** Analytic data of video game usage in sessions by gender.

Gender	Participants, n	Total app usage sessions		Journal sessions		Guided breathing activity sessions		Cognitive monsters sessions		Interaction with characters sessions	
		Mean (SD)	Range	Mean (SD)	Range	Mean (SD)	Range	Mean (SD)	Range	Mean (SD)	Range
Male	13	7.7 (12.18)	1-47	4.5 (7.48)	0-17	4.2 (5.2)	0-18	4.5 (3.54)	0-10	13.2 (14.18)	1-171
Female	17	5.1 (5.83)	1-26	3.1 (4.55)	0-19	3.1 (3.69)	0-15	8.2 (9.23)	0-38	14.1 (14.26)	0-66

Participants’ comments during the interviews shed some light on the variability observed in the use of the video game. It is possible that participants who used the game more frequently did so because it was relatable to their circumstances: “I mean, it’s pretty relatable” (Female, age 25), “…you could see areas of where it would apply to any anyone, you know?” (Male, age 72).

They also mentioned that they liked the diversity of the characters and could relate to the scenarios being presented:

Like, for example, when I was talking to the lady and she was explaining how they couldn’t have visited her for a while…her family members. It did come like it’s something that I correlate to my own story… I did come to realize that, hey you know what? It’s not me only. Different people are going through different troubles in their life under this pandemic and obviously…different people are being affected in a different way.

Male, age 28

However, participants who did not use the game very frequently thought that the content and design did not apply or seem relevant to them:

I mean, you know a lot of times it didn’t apply to me as a person. I guess I would have liked something where it was more…I know you can’t do it, but more of a live chat where I'm saying, I’m feeling this way, and then it would be more like…okay let’s see what we can do to help you, you know what I mean? So more of that versus the stock, kind of, things and stuff. Like to appeal to me as an adult, you know? And depending on the age category that this is being marketed to, I mean, if it’s a younger, yeah, it would be fine as is, totally, you know? But people that are older, like myself, have dealt with plenty as most older people have that…people as they’ve gotten older have. I just want more challenges so I really would have to think and strategize and think, okay, this is what I would do and if I want to be positive and, you know…um, and I want to conquer the negativity this is what I got to do, you know?

Female, age 54

I noticed that there was a theme just related to COVID there in it…and some of it is relatable and some of it isn’t…I think there was a bit of a limitation in that feature. Like when going back to work and putting myself and there or sending kids to school. Like those don’t apply to me so…

Male, age 28

Participants also mentioned that they enjoyed being able to select their avatars and make the avatar look like them, and they liked the diversity (n=15) of the avatar options:

I mean, that was the first thing that I did when I opened the app. Um, I really…I enjoyed that probably the most right at the beginning…I enjoyed having my little character, and it looked like me, running around the island.

Female, age 31

I really liked the diversity in the other avatars that were there. So to be able to customize my own avatar, I feel like that’s nice and like that’s a bonus. …make it a male or a female, you know, make it a different skin color, be able to wear a different color of clothes ‘cause, you know, like I feel like colors also calm me down. So if I see a lot of bright colors, I don’t like it. I like it light coloring. So, I would definitely personalize it to like a white or something like that to just, again, calm me down and like, you know, I can release my stress basically.

Female, age 25

Several participants (n=20) also mentioned they would have used the video game more often if there was more variety in the activities, if the activities were more interactive (n=10), and if there were additional levels (n=10). Participants recommended having increased complexity in the video game in terms of unlocking levels and having a sense of progression over time with the advancing levels (n=10):

Uh, but I think it could improve if there were more stress related exercises. More like that breathing exercise. That helped me… to bring down my stress, to you know, have my, um, let’s say my breath more normal. It helped me to relax but …it’s like okay, what else? I want to learn another exercise and there are no more exercises.

Age 50

The star system made no sense to me. It seemed pointless. I just need progression. I just need to continue going forward. Um, I’m not into stuff…or just like constantly battling the rabbits, getting knocked down. I think there’s eight or nine of them on that little island, and you bring them back down to normal rabbits, you turn off the app, you open it back up, and they’re all back there…kind of deterrent. So just some sort of forward progress.

Male, age 30

### Usability of the Video Game

After 2 weeks of using the video game, participants reported a mean SUS score of 73 (SD 17; median 76, IQR 43-95; N=32). Almost two-thirds of the participants (21/32, 66%) scored greater than 68 on the SUS tool, indicating a score above average. Specifically, the majority of the participants (28/32, 88%) found the video game easy to use and believed that most people can learn to use the video game quickly. The majority of the participants did not find the video game to be too complex (26/32, 82%) or cumbersome (24/32, 75%). The video game scored lower on being inconsistent to use (63%, 20/32) and being well-integrated (17/32, 53%). Participants were divided on how often they would like to use the video game; 28% (9/32) of participants reported they would like to use it frequently, 34% (11/32) disagreed with this statement, and 38% (12/32) were neutral.

When we asked participants about the usability of the video game during the interviews, the responses echoed the quantitative findings; the majority of participants reported that the video game was easy to use (28/32, 88%) and easy to learn (28/32, 88%).

However, many participants shared some functionality issues with the video game, which could explain why it scored lower in the SUS scale with being consistent to use and with being well-integrated. The most common complaint was the login process. Nineteen participants (out of the 32 we interviewed) mentioned experiencing issues with the password and login process. There was some indication the preference would be to have a login and password that could be easily remembered or saved; the experience of having to copy and paste the password limited their ease of access to the video game. Participants mentioned that immediate access would be preferable during times of stress:

…the annoying part was at…it doesn’t save your username and password…because the password is like this long thing, I had to keep copying and pasting it…and then the username, I just had to like remember it and that that like turned me off from using it. Like if it if it saved your login, that’d be much easier, like when you’re stressed, you can just click on it and go in. But then it stresses you more out to have to go into your notes on your phone and copy and paste the password.

Female, age 23

Another common complaint was not being able to find instructions on how to navigate the video game and a lack of detailed descriptions of the features:

So, for me, it would have been helpful if maybe there was some signs or labels, um, or arrows or something like that to kind of help me move around the app so I could go back to these favorite things that I I like to do, [Hmm, hmm] uh, ‘cause I found…I did get kind of lost a couple times.

Female, age 31

These complaints were expressed by participants across all age groups and genders as well as stress levels.

Another finding from the interviews was that many participants were unaware or were unable to find certain features of the video game. For example, a number of participants mentioned that they would have liked to customize some aspects of the video game, including changing the music, joystick, and timing for the breathing sphere, which were all features that were already present. Therefore, the video game settings were not visible or readily accessible to the participants.

The SUS result showing that participants were divided in how frequently they would like to use the video game was partially explained by how they perceived the design and why they were using the video game.

In terms of the design, some participants expressed that they found the design appealing (n=17) while others did not (n=15). Their like or dislike of the design was linked with how frequently they would like to use it. Of those who liked the design, they found the video game to be “creative,” “appealing to the eye,” and “liked the 80s vibe, the nostalgic, happier times.” Participants who did not like the appearance of the app remarked that it was not sophisticated, “rudimentary,” “amateur,” and not “polished.” Two participants thought that the characters needed to be more realistic in 3D and less pixelated and clunky.

I liked the cartoon aspect. …there’s a lot of different games and apps that are so focused on their graphics…that it’s just like it’s too overplayed. I liked the kind of like fantasy world. I like the cartoony stuff. It is…it just sets a different tone. You know that you are not necessarily yourself and you get like a break from, uh, reality type thing. So it’s just…I like that concept. I liked the outlook and the way everything was laid out…So it was good.

Male, age 30

But they look…I mean, I prefer a little bit more intensity in the character…just more advanced looking, you know, in terms of the character designs…they have little…kind of jaggedy. Like, because of the way it’s designed, the characters. I just prefer it being a better…better in quality, I guess…of design…it looks too…like not current, you know, in terms of design, um, and that’s the coding, I guess. I just want them to look more sophisticated and more modern, even though it’s a it’s a quaint village. It’s just the coding, uh, the programming of it…

Female, age 54

We found that participants’ experiences with the video game varied depending on their motivations for using it. For example, those who specifically sought to explore strategies for addressing stress and anxiety (n=23) found that certain features were useful or helpful in dealing with their stress, or if it was not useful, that it was a feature that could potentially be useful to others. For those who were curious or interested in the gaming aspect (n=7), we found that they did not engage with the features to any great extent and therefore did not like the features or find them to be useful.

…definitely I liked the fact that the…it is in the format of a game, uh, which I can control. Um, the other feature I really liked was how it would give me different stress coping methods or like different techniques, um, to try out and see if it can really help me with stress management…cause, as I said, you know, um, the reason of feeling stress was, you know, when you’re alone in COVID, you don’t get to go out with your friends. So I guess I was…with the use of app, I was using it as a friend, [Hmm, hmm] um, to kind of have conversations with those different, um, you know…the people that were in the app and, uh, just exploring the different areas and, you know, exploring what activities are in built in the app… But, yeah, overall, it’s a great, creative app, which will…I feel will really help out, um, people in terms of stress management.

Female, age 25

…like it was too obvious that it was, you know, um, not a game. There was, you know, very like okay, all right, this is an educational thing. This isn’t actually meant to be fun. It’s just trying to teach you something.…like I didn’t read it in depth. I was more okay with, you know, a quest for me or are you going to give me an item or something like that. So, I clicked through it and I didn’t get anything, so I think that was it. like I’m not really getting much out of this.

Male, age 23

Several participants (n=15) said they may reengage if improvements were made, including more interactions, better defined goals, and having more levels:

Well to be honest, if it is kept as is, I’ll be honest with you I don’t think so I’ll be using it much. Maybe once in a blue moon but obviously if the app is updated and like I said you have more interaction and the overall aspect feels more connected, sure like maybe even once a day I wouldn’t mind just for like two or three minutes in between breaks just to have refreshing change in scene basically.

Male, age 28

…I didn’t continue to engage because it would just say the same thing. I think that if they said something different, like depending on the day of the week, and they had like more things to say, then I would go to them...

Female, age 23

### Perceived Stress

Twenty-eight out of the 32 participants completed the PSS at baseline and follow-up. To compare the baseline PSS score to the follow-up PSS score, we conducted a paired *t* test using SPSS.

Results indicate that there was a significant difference in the participants’ PSS score at baseline compared with that at the 2-week follow-up (t_27_=3.628, 95% CI 2.785-6.430; P=.001; Table 4). The mean difference between baseline and follow-up PSS score was a decrease of 4.1 points and a medium effect size of *d* =0.67.

However, results from the question “To what extent do you think this app reduced your anxiety?” showed that most participants found the app to be neutral in reducing anxiety with a mean score of 4.59 (SD 2.769, range 0-10)

**Table 4:** Perceived Stress Scale scores at baseline and follow-up (n=28).

Time point	Mean (SD)	Range
Baseline	22.14 (6.19)	10-33
2-week follow-up	18.04 (6.08)	6-29

During the interviews, some participants thought that the video game was “great” for “winding down” and found that the video game offered a distraction (n=5), particularly from everyday life, and the gaming feature was engaging.

…because it is sort of like a distraction, if you know what I mean? ... So it was…it was useful I would say. It was actually useful too.

Male, age 39

It just gave me those couple seconds to kind of calm down, think, and not be so wound up.

Male, age 30

In the interviews, participants suggested that the “breathing sphere” had the most direct impact on their stress reduction (n=13), and that it was a skill they incorporated into their lives, even when they did not use the video game. The other features seemed to help in terms of providing a distraction.

When you breathe in and then it will shrink in size indicating when to breathe out. I think that was probably, in terms of stress reduction…that was probably the most important feature of the whole app because that was when I could feel that, okay, this is really designed to help me in terms of stress levels. And I think that was probably my favorite feature of the app as well. And, uh, I found it useful actually in in that goal as well for reducing stress. Maybe not a…maybe not too much but a little bit it did…I feel reduce a little bit of my stress.

Male, age 39

…the breathing exercise just kind of helped me focus and it did a really good job with me using that app and not being distracted with other stuff in the room and helping me slow my breathing down and take some deeper breaths. It seemed to kind of relax my body quite a bit, which was nice. … even though I wasn’t using it, what I remembered was the breathing and then…I was deepening my breathing and kind of doing slow breathing. So it was the one that impacted me the most even when I wasn’t using the app, I remembered about it and I did deeper breathing, slowing breathing. So it reduced my stress the most.

Female, age 31

While there was recognition of the cognitive restructuring and community support in the video game through combating the wererabbits and interaction with the characters, respectively, only a few participants specifically mentioned that they found it to be effective in addressing their stress. Others found it to be a fun, distracting activity.

I think one of my favorites was probably like the understanding yourself through the characters and, um, and how psychology was kind of integrated into the storyline like for example, cognitive restructuring. That’s something I knew of but I didn’t know what it was called like it just made me realize and getting to know myself better and the way I think. And putting the name to that and that was interesting. But, um, at the…in terms of the interactions themselves and, and the tips that they provide, they were useful in understanding your feelings.

Male, age 28

…the six monsters that…evolved from rabbit…and they kind of like, uh, represent…stressful ideas that you may have, uh, psychologically. So in this case, you are able to try to correct your thoughts in order to relieve the pressure. So I think in this way…I mean the steps seem very good….when I to try to fight each of the monsters into rabbits, I find that there’s actually kind of thoughts and family experience about how hard it is. But in that case those are other stressful situations but once you fight the monsters you’ll be able to find your correct thoughts I believe and they help you to relieve your stress. So those are the useful steps. I think it’s very good for young people and the older people if they are very stressful.

Male, age 25

There were also various perspectives about behavioral activation features, which included the journal and goal-setting exercises. Some participants (n=16) thought that the features were helpful and that it helped them reflect and reduce their stress:

I could look back and see what I wrote and just like, oh yeah, that was kinda crappy that day…Or maybe cause it was raining out and it minus two and I was just not happy or whatever. But, yeah. I could reflect on the past journals which was helpful. I found that more kind of helpful than maybe writing for the day although when I wrote for the day, I would write all like…like that’s where I’d write everything, so I was kinda dark. Like, I wasn’t like looking optimistic…if I was negative, I’d blurt it all out. If it was, uh…you know, if I was mad at somebody I’d blurt it all out like I didn’t hold back so that was the place to do it rather than, you know, carrying it with me. So, yeah. Oh, yeah. It did. It helped me [with stress], uh…yeah. There was stuff I wrote, and I’d look back and go yeah, okay I keep saying I’m going to do this so now I gotta do this. You know, so it challenged me. Yeah. The whole put up or shut up kinda thing. Right? Um, I found that was good [goal-setting] because I put some stuff on there that I never really have done recently in the last…not just COVID, just in my life so I thought, you know, no I’m going to write things in there that I’m going to maybe make a life changing, you know…like just to try and alter my whole mindset so, uh, yeah I found it…I mean I’m still…there’s still a couple of goals that I haven’t hit but I’m keeping them there just to, you know, maybe one day I’ll be able to get past it.

Male, age 56

Most participants who did not use behavioral activation features (the journal) thought that it could potentially be helpful to others:

So I would say I didn’t use it. I wasn’t too frequent in using it. But they were okay. Like there’s nothing wrong with them when they’re just for other people. I personally don’t use journals and like activity. Like I don’t really note things down like that…uh, like in a book or somewhere, so on a…I mean it in in one way you could say that that really didn’t attract my attention either.

Male, age 39

## Discussion

### Principal Findings

This manuscript describes the development and usability of a video game designed to help people reduce their stress during COVID-19 by gamifying CBT features. A variety of clinical recommendations were derived from the whiteboard consultations, where the results directly informed the game content, including having exercises related to behavioral activation, cognitive restructuring, mindfulness, and community supports. Further, the developers were mindful about developing a game that did not have addictive qualities, followed strict privacy requirements, did not involve an intent to commercialize (in order to maximize uptake and help as many people as possible), and was developed with inclusivity and ethical standards. Having these features in place might help with the adoption and longevity of the video game following the pandemic [34]. In particular, ensuring privacy concerns were met and that the video game was not addictive were particularly important, since several researchers have highlighted the need to enhance security and raised concerns on unhealthy use of technology in digital games related to mental health [35-37].

This study showed that most participants used the video game an average of six times, and the mean usability score was 73 on a scale of 0 to 100 as measured by the SUS. This SUS score corresponds to being “good,” as proposed by Bangor et al [38]. These findings are similar to other digital interventions addressing stress [39-42] and are encouraging, since Champion et al [42] found that improvements in stress can be achieved through short-term engagement (an average of six times) with a mindfulness-based cognitive therapy mobile app. Participants echoed some of the quantitative findings in the interviews, saying that the video game was easy to use, but mentioned a few functionality features that could be improved. They also mentioned they would have used it more often if there were more features.

In terms of stress reduction, this study showed that there was a significant difference in the participants’ PSS scores at baseline (mean 22.14, SD 6.187) compared with those at the 2-week follow-up (mean 18.04, SD 6.083; t_27_=3.628, P=.001). The mean difference between the baseline and follow-up PSS score was a decrease of 4.1 points and a medium effect size of *d*=0.67. This mean difference in PSS score is similar to the results found in other studies examining the effectiveness of mobile-based stress management apps [42-46]. Likewise, the findings from this study add to the existing array of literature that have demonstrated video games such as the Legend of Evelys to be effective in lowering psychological stress and improving mood [17,18,47-49]. Participants mentioned that using the video game distracted them from their daily stressors, and that the mindfulness exercise was particularly useful at moments of stress.

The usability results are encouraging and warrant further study on the real-world effectiveness of the video game.

### Limitations

This study had several limitations. First, the sample size for the study was small. We recruited a minimum of 3 and a maximum of 5 participants in each age and gender group, with the exception of males aged 40-50 years, where we were unable to recruit any participant. For the purpose of assessing usability of the video game, studies have demonstrated that most usability problems are detected with three to five users [50]. As a result of the small sample of participants, we were limited in our ability to stratify the findings by age, gender, and other variables of interest. Second, there was no control condition, which limits our ability to understand if any changes in the PSS score are due to using the video game rather than other factors. While we assessed participants’ self-reported stress at baseline, we did not account for any past or current COVID-19 exposure and its possible impact on stress. However, the majority of the participants reported experiencing moderate to high level of stress at baseline (25/30, 83%), which may be attributed to the impact of the pandemic on their mental health. Furthermore, we excluded individuals reporting any other psychiatric illness. It is important to note that the purpose of this study was to examine if people would use the video game and their perceptions on playing it as a stress reduction strategy. Assessing the effectiveness of the video game as a stress reduction strategy was beyond the scope of this study. Third, it should be noted that 66% (21/32)of participants were single (never married), which is higher than the case reported in Ontario’s 2016 census [51]. Similarly, compared to the 2016 census [51], a larger proportion of participants in this study had a college or university degree. Another limitation is the limited period that the video game was tested by each participant; however, given that the video game had a short storyline and only four CBT features to test, the time was likely of sufficient length.

### Conclusion

Through this collaboration and development of the video game, we have established that it is possible to gamify CBT exercises to address stress while also establishing an inclusive, secure, and ethical design that is inclusive of users with different abilities. Participants used the video game an average of six times over a span of 2 weeks. The findings demonstrated good usability of the video game and significant reduction in users’ perceived stress within this short time frame. While we recognize video games as a promising strategy to help people with their stress and anxiety, we have also identified several limitations of the study that limit our ability to fully understand its impact on stress reduction. As a next step, further study is needed to examine the real-world effectiveness of the Legend of Evelys in reducing anxiety.

## Appendix

Multimedia Appendix 1 Screenshot of the Legend of Evelys app showcasing user interactions with characters in the video game.

Multimedia Appendix 2 Screenshot of the Legend of Evelys app showcasing the cognitive restructuring activity where users encounter a fight with a “cognitive monster”.

## Abbreviations

CAMH: Centre for Addiction and Mental Health
CBT: cognitive behavioral therapy
JSON: JavaScript Object Notation
NRC: National Research Council of Canada
PSS-10: 10-item Perceived Stress Scale
SUS: System Usability Scale
