# Supplementary figures and images for: Video Game to Attenuate Pandemic-Related Stress From an Equity Lens: Development and Usability Study
Source: JMIR Form Res. 2022 May 12;6(5):e36820. doi: 10.2196/36820 (PMC9136657; doi:10.2196/36820)

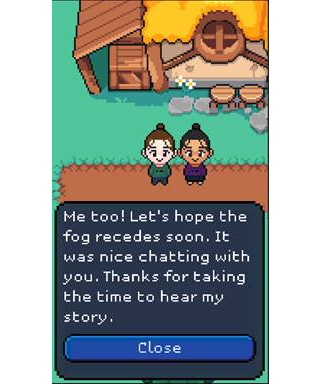

Supplement: Multimedia Appendix 1 [file formative_v6i5e36820_app1.png]

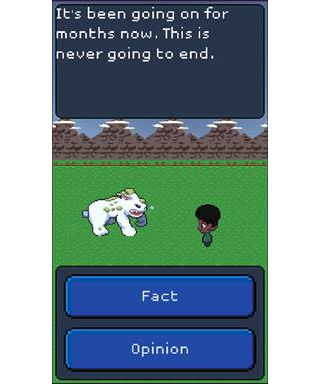

Supplement: Multimedia Appendix 2 [file formative_v6i5e36820_app2.png]
